# Different processes lead to similar patterns: a test of codivergence and the role of sea level and climate changes in shaping a southern temperate freshwater assemblage

**DOI:** 10.1186/1471-2148-11-343

**Published:** 2011-11-25

**Authors:** Brian R Barber, Peter J Unmack, Marcos Pérez-Losada, Jerald B Johnson, Keith A Crandall

**Affiliations:** 1 Evolutionary Ecology Laboratories, Department of Biology, Brigham Young University, Provo, UT 84602 USA; 2 CIBIO, Centro de Investigação em Biodiversidade e Recursos Genéticos, Universidade do Porto, Campus Agrário de Vairão, 4485-661 Vairão, Portugal; 3 Monte L. Bean Life Science Museum, Brigham Young University, Provo, UT 84602 USA

**Keywords:** *Aegla*, biogeography, Chile, cladogenesis, freshwater, *Trichomycterus*

## Abstract

**Background:**

Understanding how freshwater assemblages have been formed and maintained is a fundamental goal in evolutionary and ecological disciplines. Here we use a historical approach to test the hypothesis of codivergence in three clades of the Chilean freshwater species assemblage. Molecular studies of freshwater crabs (*Aegla*: Aeglidae: Anomura) and catfish (*Trichomycterus arealatus*: Trichomycteridae: Teleostei) exhibited similar levels of genetic divergences of mitochondrial lineages between species of crabs and phylogroups of the catfish, suggesting a shared evolutionary history among the three clades in this species assemblage.

**Results:**

A phylogeny was constructed for *Trichomycterus areolatus *under the following best-fit molecular models of evolution GTR + I + R, HKY + I, and HKY for cytochrome *b*, growth hormone, and rag 1 respectively. A GTR + I + R model provided the best fit for both 28S and mitochondrial loci and was used to construct both *Aegla *phylogenies. Three different diversification models were observed and the three groups arose during different time periods, from 2.25 to 5.05 million years ago (Ma). Cladogenesis within *Trichomycterus areolatus *was initiated roughly 2.25 Ma (Late Pliocene - Early Pleistocene) some 1.7 - 2.8 million years after the basal divergences observed in both *Aegla *clades. These results reject the hypothesis of codivergence.

**Conclusions:**

The similar genetic distances between terminal sister-lineages observed in these select taxa from the freshwater Chilean species assemblage were formed by different processes occurring over the last ~5.0 Ma. Dramatic changes in historic sea levels documented in the region appear to have independently shaped the evolutionary history of each group. Our study illustrates the important role that history plays in shaping a species assemblage and argues against assuming similar patterns equal a shared evolutionary history.

## Background

Understanding how species assemblages have been formed and maintained is a central question in biology [1-4]. Historical processes, such as river capture and climate change, likely played significant roles in driving the formation of aquatic species assemblages [5-7], whereas ecological processes may help maintain species interactions in co-occurring species if in fact those species are interacting (i.e form a community). Understanding the relative roles these two processes have had in shaping species assemblages or communities is challenging [8]. A logical first-step, especially in poorly studied regions, is to apply historical methods to understand how past events have shaped current patterns [9]. In this study we apply comparative phylogeography and biogeography methods [10,11] to test the hypotheses that historical abiotic events are responsible for generating the pattern of similar levels of genetic divergence recently observed in two diverse taxa (three clades) that are codistributed in freshwater systems of Chile.

**Figure 1:**
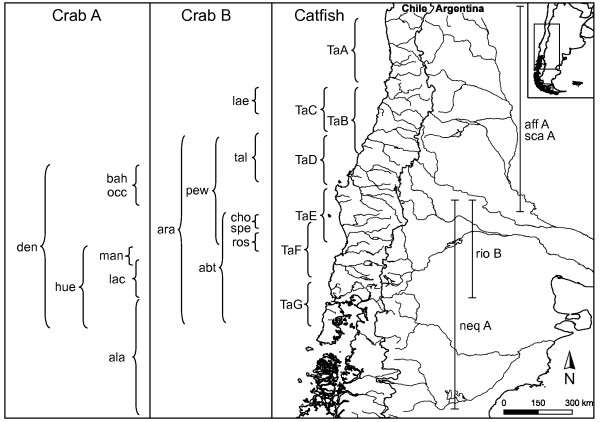
**Map of South America showing major river systems of Chile and Argentina and distributions of *Aegla *taxa and *Trichomycterus areolatus *clades**.

Freshwater systems in Chile are biogeographically unique and were likely impacted by changes in sea levels (Figure 1). High gradient rivers originate in the Andes Mountains (1000 - 7000 m above sea level) then flow westward to the Pacific Ocean, a distance of only 100 - 220 km [12]. Currently, most Chilean rivers are quite isolated from each other with the most likely colonization routes between them are across drainage divides or via coastal seas [13]. Gene flow between drainages could have occurred if rivers coalesced on the continental shelf during historical periods of low sea levels. However, the continental shelf is narrow, only 10 - 60 km, providing limited opportunity for rivers to coalesce during lowered sea levels [13]. We predict divergence between populations would occur when sea levels are high, because the already restricted freshwater systems would have been truncated even more, thereby facilitating genetic drift and the accumulation of genetic divergence. In contrast, incipient divergence would have been erased when admixture occurred during lowered sea levels because previously isolated populations were merged. Overall, dramatic fluctuations in sea level should alter cladogenic rates. In addition, Late Pleistocene climate changes might have altered cladogenic rates due to changes in stream flow patterns and volume that altered the distribution of these organisms.

**Figure 2:**
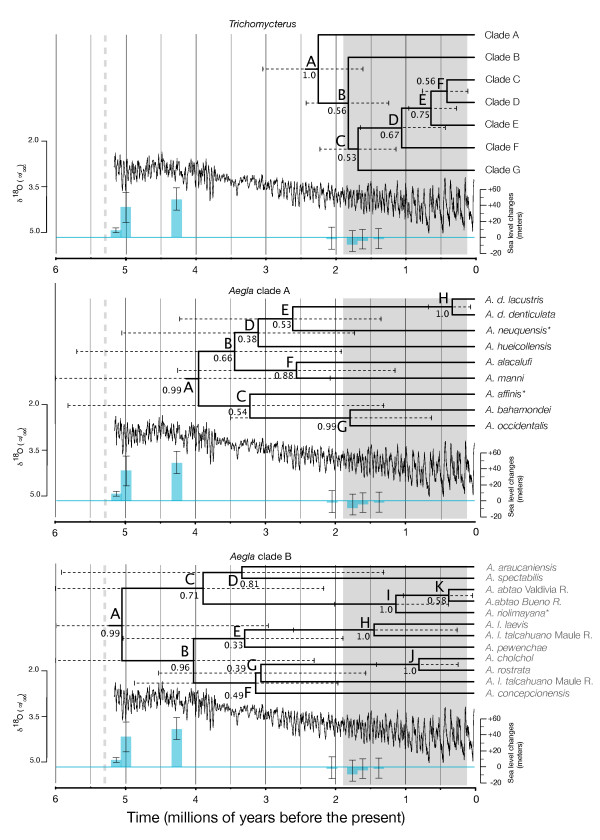
**Species trees of three Chilean freshwater taxa, climate change and major episodes of sea level changes documented over the last 6 million years**. Sea level changes (in blue, with max/min-bars) estimated from the Bay of Tongoy, Chile (Figure 1;[24]). Jagged lines indicate climate change inferred from benthic δ^18^O obtained from 57 globally distributed sites [25]. Pleistocene period indicated with gray background. Lower boundary of the Miocene epoch showed with a vertical dashed line. Species of *Aegla *with asterisks occur in Argentina. Letters refer to nodes discussed in the text and table 1. Posterior probabilities for nodes are below or next to node labels. Horizontal dashed lines are the 95% HPD estimates of the node dates.

Compared to the Northern Hemisphere, relatively few phylogeographic or phylogenetic studies have been undertaken in southern latitudes, especially in temperate freshwater ecosystems [14]. However, phylogeographic and phylogenetic studies of terrestrial and freshwater taxa in Chile and Argentina [15,16] are beginning to provide insights into the evolutionary history of this important region. Because aquatic diversity in Chile is relatively depauperate [17,18] examination of a few widespread can provide important insights into the evolutionary history of the region. For example, a phylogenetic study of freshwater crabs in the genus *Aegla *(Aeglidae: Anomura; [19,20] and a phylogeographic study of the freshwater catfish (*Trichomycterus areolatus*: Trichomycteridae: Teleostei; [13] discovered similar levels of model-corrected genetic distance of mitochondrial lineages between *Aegla *(cytochrome oxidase I and II genes: 1598 base pairs) and *Trichomycterus *(cytochrome b: 1137 base pairs). For example, genetic distance between two rivers, Río Maipo and Río Maule, are 2.6 - 2.8% and 2.0 - 2.8% in *Aegla laevis *and *Trichomycterus areolatus *respectively. In addition, genetic distances between codistributed sister-lineages are similar. For example, sister-clades (phylogroups: D + E: Figure 2) in *Trichomycterus areolatus *and sister-taxa *Aegla bahamondei *+ *A. occidentalis *have overlapping divergence values of 1.0 - 2.3% and 1.7% respectively. Two codistributed phylogroups in *Trichomycterus areolatus *(clades F + G) and within *Aegla abtao *have similar, but non-overlapping, divergence values of 3.3 - 4.8% and 2.4 - 3.2% respectively (see [13] for further discussion). Similar levels of genetic distance between contemporary lineages suggest these taxa may have been influenced by the same events. Finally both taxa are codistributed and often sympatric in the region suggesting both may have been similarly affected by the abiotic events that have shaped the region [13,20-23].

In this study we test the hypothesis of codivergence between *Aegla *species and *Trichomycterus *phylogroups. In other words, are the same historical events documented in the region responsible for generating the nearly identical levels of genetic divergence observed in these taxa? Four major geological and climate events may have played a role in shaping the evolutionary history of these taxa. First, the establishment of freshwater systems in the region that were precursors to current day drainages, some nine million years ago (Ma), may have driven initial lineage splitting [12,21]. Second, two dramatic fluctuations in sea-levels (~5.0 Ma and ~2.0 Ma: [24]) documented in the region might have altered cladogenic rates. Finally, the relatively high amplitude climate oscillations experienced during the last million years of the Pleistocene might have increased or decreased speciation rates [25,26]. We generate specific predictions on the type of diversification models expected based on the impact of these abiotic events (Table 1). Furthermore we predict that if these shared events shaped genetic patterns across codistributed taxa, we expect to detect identical cladogenic rates and similar dates of lineage splitting in *Aegla *and *Trichomycterus*. To this end we examine the fit of six models of diversification to the catfish and crab phylogenies. In addition, we determined splitting times for major nodes. We reject the hypothesis of codivergence if both models of diversification and times of divergence differ across taxa.

**Table 1:** Hypothesized outcomes on cladogenic rates caused by major geological and climatic events documented in the region.

Abiotic event	Time period	Predictions and expectations	Reference
Establishment of freshwater systems in Chile	~ 9.0 Ma	Origins and initial diversification Basal nodes date to this period	[12,21]
Dramatic increase in sea-levels	4.3 - 5.3 Ma	Increased cladogenic events Significant shifts in cladogenic rates (Yule two rate or rate-variable birth-death)	[24]
Dramatic decrease in sea-levels	1.4 - 2.2 Ma	Decreased cladogenic events Significant shifts in cladogenic rates (Yule two rate or rate-variable birth-death)	[24]
Late Pleistocene climate oscillations (Driver of speciation)	1.0 - 0.1 Ma	Increase in cladogenic events Majority of cladogenic events date to this period & Significant shifts in cladogenic rates (Yule two rate or rate-variable birth-death)	[25,26]
Late Pleistocene climate oscillations (Increased extinction)	1.0 - 0.1 Ma	Decline in cladogenic rates Few cladogenic events date to this period & Decline in cladogenic rates (density dependent model [logistic or exponential] of diversification)	[25-27,53]
No impact	Throughout	No change in cladogenic rates Constant cladogenesis models: yule pure-birth or pure-birth	

## Results

### Dates of cladogenic events

We found that our three taxonomic groups originated during different time periods (Table 2 and Figure 2). Cladogenesis within *Trichomycterus areolatus *was initiated roughly 2.25 Ma (Late Miocene - Early Pliocene) some 1.7 - 2.8 million years after the basal divergences observed in both *Aegla *clades. The 95% highest posterior density (HPD) of the basal *Aegla *nodes are consistant with episodes of increased sea levels documented at the Bay of Tongoy, Chile (~ 4.2 - 5.0 Ma; [24]), whereas the basal node of *Trichomycterus areolatus *emerge during a time of decreased sea levels that occurred in the region roughly 1.4 - 2.2 Ma [24]. Both crab phylogenies date to the Pliocene with *Aegla*A basal divergence occurring most recently (3.95 Ma), whereas *Aegla*B originated 5.05 Ma. Moreover, the majority of cladogenic events (15/19) in the crab phylogenies date to the Pliocene or Early Pleistocene (> 1 Ma). Only three cladogenic events, (*Aegla*A: node H; *Aegla*B: node J and K) occurred during the especially high amplitude climate cycles of the last 1 million years. All but one of the six cladogenic events in *Trichomycterus areolatus *occurred during the Pleistocene, with two events consistent with the high amplitude climate cycles of the last 1 Ma. Overall only five of the twenty-five cladogenic events events occurred during the last 1 Ma

**Table 2:** Estimates of the ages of major nodes in the *Trichomycterus areolatus *and *Aegla *phylogenies (Figure 2).

Node	*Trichomycterus*	*Aegla *A	*Aegla *B
A	2.25 (1.54-3.09)	3.95 (2.16-*6.56*)	5.05 (2.94-*7.84*)
B	1.82 (1.23-2.43)	3.44 (1.83-*5.70*)	4.02 (2.33-*6.17*)
C	1.68 (1.15-2.24)	3.22 (1.39-*5.78*)	3.89 (2.17-*6.09*)
D	1.05 (**0.45-**1.66)	3.10 (1.58-5.11)	3.33 (1.36-*5.81*)
E	**0.62 **(**0.36-0.92**)	2.60 (1.33-4.23)	3.30 (1.80-5.07)
F	**0.39 **(**0.21-0.61**)	2.55 (1.17-4.23)	3.13 (1.61-4.81)
G		1.78 (**0.62-**3.50)	3.06 (1.94-4.57)
H		**0.32 **(**0.07-0.63**)	1.44 (**0.37**-2.62)
I			1.12 (**0.32**-2.03)
J			**0.79 **(**0.24**-1.36)
K			**0.37 **(**0.01**-1.07)

Mean estimates and 95% HPD are given in millions of years before present. Values in bold fall within the last 1 Ma, italics designates dates that are Miocene origin, all others are Pliocene or Early Pleistocene (> 1 Ma) origin.

Effective sample sizes for node estimates were over 500 (as were most parameters); thus, we assume the results are from thoroughly sampled analyses. Models selected using AIC for *Trichomycterus areolatus *were GTR + I + R, HKY + I, and HKY for cyt-*b*, gh, and rag1 respectively. The GTR + I + R model provided the best fit for both 28S and mitochondrial loci for both of the *Aegla *datasets. Nuclear mutation rates estimated from the analyses are as follows: *Trichomycterus areolatus *(rag1 = 4.48 × 10^-4 ^[2.34 × 10^-4 ^- 6.79 × 10^-4^] and gh = 1.24 × 10^-3 ^[7.41 × 10^-4 ^- 1.81 × 10^-3^]), *Aegla*A (28S = 1.63 × 10^-3 ^[7.25 × 10^-4 ^- 2.58 × 10^-3^], *Aegla*B (28S = 1.04 × 10^-3 ^[5.38 × 10^-4 ^- 1.60 × 10^-3^].

### Diversification tests

The best-fitting model of diversification (Table 3) for *Trichomycterus *was pure-birth (delta AICrc = 1.36, p = 0.21), whereas the logistic density dependent model provided the best fit for *Aegla*A (delta AICrc = 7.79, p = 0.02). A rate-variable birth-death (rvbd) model was the best model for *Aegla*B (delta AICrc = 5.47, p = 0.03).

**Table 3:** Results of maximum likelihood tests of diversification models.

Taxon	Best constant model	Best variable model	ΔAICrc
*Trichomycterus*	**pure birth (AIC = 5.82)**	DDL (AIC = 4.47)	1.36 (p = 0.21)
*Aegla *A	pure birth (AIC = 12.56)	**DDL (AIC = 4.77)**	7.79 (p = 0.02)
*Aegla *B	pure birth (AIC = 11.79)	**rvbd (AIC = 6.32)**	5.47 (p = 0.03)

Shown are best constant and best variable rate models selected from six candidate models. Tests of significance were conducted by simulating 5000 phylogenies under a constant birth-death model. The best constant model was rejected if the ΔAICrc was significant (two-tailed alpha = 0.05,[49]). DDL is a logistic density dependent model and rvbd is a rate-variable birth-death model. The best-fitting model is in bold.

## Discussion

This study was motivated by the observation of similar levels of genetic distances between lineages in three clades that are codistributed in freshwater systems of Chile. Our goal was to determine if this pattern was the result of codivergence driven by historical abiotic events. We discuss the temporal origins of the three groups. Then we discuss results of diversification tests and mechanisms that are postulated to have shaped this assemblage of freshwater taxa.

### Temporal origins

Lineage splitting time (mean) estimates of basal nodes vary considerably (2.18 - 4.50 Ma), suggesting different processes generated initial cladogenesis (Table 2 and Figure 2). The confidence intervals of the two crab groups overlap considerably, suggesting similar temporal origins. However, different models of cladogensis were recovered for these two groups suggesting different processes have shaped these two groups (see next section). The hypothesis that the original formation of river systems in the region some 9 Ma [12,21] played a role in shaping this fauna is rejected. However the exceptionally high sea levels that occurred from 5.0 to 4.2 Ma are consistent with the origins of both *Aegla *clades (Table 2 and Figure 2; [24]). Cladogenesis within *Trichomycterus areolatus *occurred relatively recently (~2.25 Ma) and is consistent with a period of low sea levels. This time period is also consistent with the second set of cladogenic events observed within *Aegla*B. The majority of cladogenic events within *T. areolatus *appear in the Pleistocene with the last two events dating to a period (< 1 Ma) of high amplitude climate oscillations (Table 2 and Figure 2). Only three cladogenic events occurred during the last 1 Ma in the two crab clades. The relatively few cladogenic events observed during the last 1 Ma reject the hypothesis that the especially high amplitude climate cycles of this period played a significant role in driving cladogensis within these taxa [26].

### Models and mechanisms of cladogenesis

Three different models of diversification were supported rejecting the hypothesis of codivergence among taxa. The pure birth model of diversification best explains cladogenesis within *Trichomycterus areolatus *and suggests that the rate initiated in this taxon was maintained throughout its evolutionary history and argues against any significant impact on rates by later sea-level changes or climate oscillations. A density-dependent model of declining diversification is the best explanation of cladogenesis in *Aegla*A.

Declines in diversification rates can occur for several reasons, but are most notable in adaptive radiations [27,28]. Speciation rates can decline as available niches become filled with the accumulation of species [29] or if an underparameterized model of molecular evolution is employed [30]. The molecular models employed in this study are relatively complex suggesting the rate decline is not an artifact of the analysis. Alternatively, the rate decline could be due to increased extinction rates.

To our knowledge, no empirical data exist on population sizes in *Aegla*, thus we can only make broad assumptions on the relative sizes of populations and their susceptibility to extinction. The distributions of several species of Chilean *Aegla *are restricted to single or several adjoining river system suggesting small populations [19]; Figure 1). Restricted and fragmented distributions coupled with small isolated populations in some species [16] suggest that population sizes are small, possibly increasing the risk of extinction [31]. In addition, population sizes, inferred from estimates of genetic diversity, are low and declining in one crab species, *Aegla alacalufi *[16].

Alternatively, especially high amplitude climate cycles over the last one million years may have increased extinction. Only one lineage splitting event dates to the Late Pleistocene (< 1 Ma) rejecting the hypothesis that this period was a driver of speciation. Rather the decline in diversification suggests that the Late Pleistocene climate cycles, or the lowered sea levels of ~ 2 Ma, may have inhibited speciation or increased extinction within *Aegla*A. A phylogeographic study of *Aegla alacalufi *recovered deeper divergence between non-glaciated populations than between glaciated populations [16]. Many of the drainage systems inhabited by Chilean *Aegla *species were covered at least partially by ice during the last and presumably previous glacial cycles [13,16]. It is likely then that repeated glacial cycles coupled with small population sizes in many *Aegla *species inhibited diversification by increasing extinction rates.

The rate-variable model best explains diversification in *Aegla*B and suggests that this clade experienced a more dynamic evolutionary history than the other clades. That is the onset of cladogenesis initiated approximately 5 Ma were later altered by abiotic events. A second cluster of cladogenic events begins 1.44 Ma (node H) which is a time consistent with both lowered sea levels and the onset of high amplitude glacial cycles, suggesting that these these events increased diversification. Thus in contrast to *Aegla*A and *Trichomycterus areolatus *this period is postulated to be a driver of cladogenesis in *Aegla*B.

### Tests of codivergence

Historical approaches have been used extensively in the study of codivergence in host-parasite systems [32]. For example, the assumption that pattern equaled process in hantavirus was assumed because the virus phylogeny formed three primary clades that coincided with the three rodent subfamilies that formed their hosts and the fact that there is a high degree of host specificity in the system [33]. However, [33] were able to reject the hypothesis of codivergence between the hantavirus virus and their mammalian hosts.

In this study historical methods were employed to determine if abiotic events were possibly responsible for driving diversification in these clades, which in turn allows us to test the hypothesis of codivergence. The observation of similar levels of genetic distance between major lineags among these taxa suggests that they are the result of either shared evolutionary or ecological processes. Our study illustrates the important role that history can play in shaping current patterns and argues against assuming that the observation of similar patterns among codistributed taxa equals a shared history [9]. It remains to be determined how, if any, ecological interactions have shaped this species assemblage.

Employing phylogeographic and phylogenetic methods to test hypotheses of codivergence among contemporary faunas is an exciting and developing research area [34]. A fundamental challenge in this endeavor will be choosing methods that can tease apart complex evolutionary histories among taxa. In this paper we employed two different methods to test the hypothesis of codivergence among our three focal groups. Obviously there are some caveats with these methods. For example, confidence intervals on divergence dates are wide and in some cases overlap multiple abiotic events and divergence times from the other taxa. Relying on this method alone would not have allowed us to test our hypothesis. However, differences in timing of lineage splitting combined with the fact that we observed three taxon specific models of cladogenesis allows us to reject the hypothesis of codivergence. Another potential problem arises by using mutation rates that are not taxon specific. This is an issue for most comparative molecular studies due to the paucity of taxon specific rates. Because mutation rates vary widely across taxa - vertebrates versus invertebrates in our study - assuming a common rate is an unacceptable solution. Finally we recognize our relatively taxon poor phylogenies may make it difficult to rigorously compare models of diversification [27,35]. For example missing or undiscovered taxa could cause an increase, decrease or shift in diversification rates. The previous phylogeographic and phylogenetic studies of these two taxa did recover undescribed independently-evolving lineages and these were included in this study. Despite these caveats our study illustrates the potential to test codivergence among codistributed fauna when multiple approaches are employed [34,36-39]

## Conclusions

Results from two different methodological approaches reject the hypothesis of codivergence among our three focal groups. Rather these representatives of the Chilean freshwater assemblage evolved through temporally-independent processes. Although this freshwater fauna is relatively depauperate additional taxa need to be studied to determine if this is a pervasive observation in freshwater systems of Chile and how ecological interactions are currently operating on the entire species assemblage. This study illustrates the importance of using historical methods to test hypotheses on how contemporary patterns have been formed and cautions against assuming that congruent patterns are the result of shared processes or ecological interactions alone.

## Materials and methods

### Chilean taxa and molecular data

The catfish *Trichomycterus areolatus *and 21 species of *Aegla *crabs were studied from the southern temperate freshwater river systems of Chile (Figure 1). *Trichomycterus areolatus *is an abundant and widespread freshwater fish with broad ecological tolerances [13]. Data for *Trichomycterus areolatus *are the mitochondrial cytochrome *b *gene (1137 base-pairs, [bp] GenBank accession numbers FJ772091 - FJ772237) and two newly obtained nuclear loci (growth hormone [1491 bp: JN186609 - JN186714] and rag1 [992 bp: JN186409 - JN186531]) from 63 individuals comprising seven clades [13]. Like the catfish, Chilean *Aegla *crabs are widely distributed and occur in a variety of freshwater habitats [23]. Two non-sister clades of mostly Chilean *Aegla *species were examined [19,23]. The first clade, hereafter referred to as *Aegla*A, includes nine taxa, the second, *Aegla*B, consists of twelve taxa [23]. Molecular data for *Aegla *consisted of the cytochrome oxidase I and II genes (1598 bp: AY049985-050166) and the nuclear 28S gene (3105 bp: AY595931 - AY595973, AY596092 - AY596093), and 20 and 24 individuals from *Aegla*A and *Aegla*B, respectively [19,20,23].

### Phylogeny and dating

The best-fitting model of molecular evolution for each locus was determined using the Akaike Information Criterion (AIC) in jModeltest [40]. A species tree and dates of major nodes were estimated simultaneously using *BEAST [41] as implemented in Beast 1.6.1 [42]. A Yule tree prior, piecewise linear and constant-root population size model and lognormal molecular clock were assumed. Analyses were run 5.0 × 10^7 ^generations with a burn-in of 10% [43]. We assumed a mitochondrial mutation rate of 1.0 × 10^-8 ^[44], and a range of 8.3 × 10^-9^- 1.3 × 10^-8 ^[45,46] for *Trichomycterus areolatus *and *Aegla*, respectively. Mutation rates for all nuclear markers were estimated, relative to the mitochondrial rate, using a normal prior. Due to uncertainty in the appropriate outgroup a midpoint root was used to root the *Trichomycterus areolatus *phylogeny [13]. Two individuals of *Aegla papudo *(cytochrome I: AY050077 - AY050078; cytochrome II: AY050121-AY050121; 28S: AY595929 - AY595930) were used to root the *Aegla *phylogeny [19,20,23]. Mean divergence estimates in millions of years and their 95% confidence intervals were obtained for all nodes using TRACER v1.4 [47]. If dates of major nodes or their confidence intervals overlap with abiotic events (i.e., sea level changes; Table 1), we assume those events may have been responsible for driving divergence at those nodes [48].

### Tests of diversification models

The hypothesis of codivergence predicts the same process of cladogenesis should be observed across taxa. To this end we evaluate the fit of six explicit models of diversification to each of the three phylogenies using a maximum likelihood test implemented in LASER [49]. In general, these models provide the means to determine if cladogenic rates were altered (i.e. by abiotic events), or remained constant (no effect), during the phylogenetic history of each group. If these taxa codiverged, due to shared historical events, we expect each to have evolved under the same model. The six models compared were: Yule pure-birth, birth-death, logistic density-dependent, exponential density-dependent, Yule two-rate, and a rate-variable birth-death model. These models capture expected patterns of cladogenesis under various historical scenarios (Table 1). For example, if changes in Pliocene sea levels altered rates, we expect to observe an instantaneous shift in rates. Similarly, we would observe a shift in rates if Late Pleistocene climate change increased lineage splitting. Under these two scenarios, we expect to observe either a Yule two-rate or rate-variable birth-death model. In contrast, if Late Pleistocene climate change decreased diversification, through increased extinction or decreased speciation, we would expect to detect either a logistic or exponential density dependent model. Finally, constant models (Yule pure-birth or birth-death) are the expected outcome if climate and sea level changes had no impact on speciation rates (pure-birth and birth-death models assume no extinction has occurred). The best constant (RC) and best rate variable (RV) model were determined using (AIC). Delta AICrc (= AIC_RC _- AIC_RV_) was used as a test statistic. Significance of each delta AICrc was determined using null distributions derived from 5,000 trees simulated under a constant birth-death (extinction = lineage splitting) model (two-tailed alpha = 0.05, [49]).

Our objective in this study was to investigate processes of cladogenesis in codistributed taxa with similar genetic distances between evolutionarily distinct lineages. Unmack *et al *[13] phylogeographic study of *Trichomycterus areolatus *recovered seven well-supported terminal clades with little to no geographical overlap between them. These clades had model-corrected sequence divergences between them greater than many recognized species (> 2%: e.g. [11,50]). These observations qualify the seven clades as evolutionarily distinct lineages under several concepts including evolutionarily significant units (ESU: [51]) and the phylogenetic species concept [52]. Therefore, we recognized each of the seven clades recovered in *Trichomycterus areolatus *as taxonomically equivalent to the currently recognized species as well as several recently discovered lineages in *Aegla*.

## Authors' contributions

BRB and JBJ conceived the study. BRB conducted analyses and wrote the paper. BRB and PJU created figures. PJU, MPL, KAC and JBJ generated data. All authors read and approved the final manuscript.
